# Monitoring of B Cell in Kidney Transplantation: Development of a Novel Clusters Analysis and Role of Transitional B Cells in Transplant Outcome

**DOI:** 10.3390/diagnostics11040641

**Published:** 2021-04-01

**Authors:** Rafael Alfaro, Isabel Legaz, Gema González-Martínez, Víctor Jimenez-Coll, Helios Martínez-Banaclocha, José Antonio Galián, Carmen Botella, Jesús de la Peña-Moral, María Rosa Moya-Quiles, José Antonio Campillo, Alfredo Minguela, Santiago Llorente, Manuel Muro

**Affiliations:** 1 Immunology Services, University Clinical Hospital Virgen de la Arrixaca-Biomedical Research Institute of Murcia (IMIB), 30120 Murcia, Spain; raf.hellin@gmail.com (R.A.); victorjcoll@gmail.com (V.J.-C.); heliosmar@live.com (H.M.-B.); diegoarmandogalian@hotmail.com (J.A.G.); carmen.botellam@gmail.com (C.B.); rosa.moya2@carm.es (M.R.M.-Q.); josea.campillo@carm.es (J.A.C.); alfredo.minguela@carm.es (A.M.); 2 Department of Legal and Forensic Medicine, Biomedical Research Institute (IMIB), Regional Campus of International Excellence “Campus Mare Nostrum”, Faculty of Medicine, University of Murcia, 30100 Murcia, Spain; isalegaz@um.es (I.L.); gemagm89@gmail.com (G.G.-M.); 3 Pathology Services, University Clinical Hospital Virgen de la Arrixaca-Biomedical Research Institute of Murcia (IMIB), 30120 Murcia, Spain; jmpc1@um.es; 4 Nephrology Services, University Clinical Hospital Virgen de la Arrixaca-Biomedical Research Institute of Murcia (IMIB), 30120 Murcia, Spain; sllorentev@telefonica.net

**Keywords:** acute rejection, B cell clusters, kidney transplant, forensic pathology

## Abstract

Background: B lymphocytes (BL) seem to play an important role in transplantation, although the and role of different subpopulations in monitoring and outcome is not clear. Our aim was to monitoring immunological profiles based on BL subpopulations in kidney recipients (KR) with the risk of acute rejection (AR). Methods: Monitoring of BL subpopulations was performed by flow cytometry in PBLs before transplantation and three and six months after transplantation (PTX). We used two methodological approaches, a traditional analysis, and a novel cluster analysis, to determine the association between BL subpopulations, AR incidence, and graft function. Results: After three months of PTX, KRs with a B phenotype enriched in transitional BL and plasmablasts had better kidney function and lower AR incidence. KRs with decreased transitional BL and plasmablasts were associated with lower kidney function and higher AR PTX. KRs that had an increase in transitional BL PTX had a better clinical outcome. The increase in transitory BL during PTX was also associated with an increase in Tregs. Indeed, KRs receiving thymoglobulin as induction therapy showed a slight decrease in the relative frequency of naive BLs after three months of PTX. Conclusion: The monitoring of BL subpopulations may serve as a non-invasive tool to improve immunological follow-up of patients after kidney transplantation. However, further studies are needed to confirm the obtained results, define cut-off values, and standardize more optimal and even custom/customized protocols.

## 1. Introduction

B lymphocytes (BL) play an essential role in the biological processes mediating renal graft rejection and tolerance through various effector mechanisms, mainly through the production of antibodies and the presentation of antigens to T cells, as well as the secretion of cytokines [1]. In the early stages, the still immature B cells that have just been released from the bone marrow are called transitional B cells [1,2]. These BLs are characterized by IgM and IgD expression, by the surface markers CD24++ CD38++ and the absence of the memory marker CD27. Already in the periphery, BLs mature into naive cells with the phenotype CD24+CD38+CD27−. In response to stimulation with specific antigens, BLs differentiate into plasmablasts, plasma cells, or memory BLs [1]. The main types of B cells are shown in Table 1.

Plasma cells are long-lived differentiated cells whose function is antibody production. These migrate to the bone marrow, where they continue to produce antibodies to protect against re-infection. The memory B response is more potent to antigens than the primary B responses and produces responses with greater affinity and immunoglobulin recombination [1,3,4].

BLs can also secrete cytokines that are pro- and anti-inflammatory, modulating the T cell response [3]. Animal studies have shown that naive BLs can differentiate into different cytokine-producing effector subpopulations depending on Th1 or Th2 cells [1].

Recent studies have also shown that BLs are not mere plasma cell precursors but that their impact on tolerance and graft rejection processes being much more complicated [1,4]. Specific subpopulations of BLs with immunosuppressive capabilities called regulatory B cells (Breg) have been shown to play an important role in tolerance by inhibiting effector responses.

Breg cells are a minority population that can arise at various stages of BLs maturational development being abundant within the transitional B, memory B, and plasmablast subpopulations [4].

As there is no consensus on their definition and classification, Bregs are defined from a functional perspective as IL-10 producing B cells, as this cytokine plays a central role in immunosuppressive functions. Despite this definition, Bregs may also exert their function through other mechanisms independent of IL-10, such as secretion of IL-35, TGF-β, or through cell–cell contacts.

Experimental data suggest that the Breg development can be induced in the periphery in response to various signals, such as BCR signaling, cytokines, or costimulatory signals [5]. Other stimuli, such as proliferation-inducing ligand (APRIL), have also shown their ability to induce Breg through its binding to its receptor.

Similarly, stimulation by CD40 has been shown to increase the immunosuppressive capacity of transitional B cells in vitro [6]. On the other hand, signaling mediated by Toll-like receptors (TLR) induces the production of IL-10 producing BLs. Mice deficient in TLRs signaling pathway proteins, such as MYD88, cause chronic inflammation due to the inability of Breg to control T cell-mediated responses [7].

The effector mechanisms by which Breg mediates immune suppression can be divided into two broad groups, IL-10 dependent or independent mechanisms. These mechanisms can act directly by inhibiting Th1 and Th17 cell responses and indirectly by promoting Treg proliferation, by increasing expression of the transcription factor FOXP3 [6]. Breg appears to play an essential role in transplant tolerance. Bregs have been associated with beneficial graft function and a lower risk of donor-specific antibody (DSA) production. In this sense, operational tolerant patients show an increase in IL-10 producing B cells, and tolerant recipients show an increase in granzyme B producing B cells, which inhibit T cell activation and suppress proliferation via apoptosis through mechanisms that are dependent on granzyme B and independent of soluble factors such as IL-10 or TGF-β [8]. In non-tolerant transplant recipients, increased Bregs levels were associated with prolonged graft survival and fewer rejection episodes [9,10]. These results suggest that these cells can be used as prognostic biomarkers of good kidney function. Breg cells are also capable of secreting pro-inflammatory cytokines such as TNF-α [1]. It was observed that transplant patients with stable renal function have higher IL-10: TNF-α ratios than those with chronic rejection. Lower IL-10:TNF-α ratios are correlated with poorer long-term outcomes as they are functionally less effective in suppressing Th1 responses [10].

Thus, we aimed to investigate Breg and the BL subpopulations in which it is enriched, such as transitional BLs, as a potential tool for monitoring kidney transplants.

## 2. Material and Methods

### 2.1. Demographic Data, Clinical Characteristics, and Study Design

**Figure 1:**
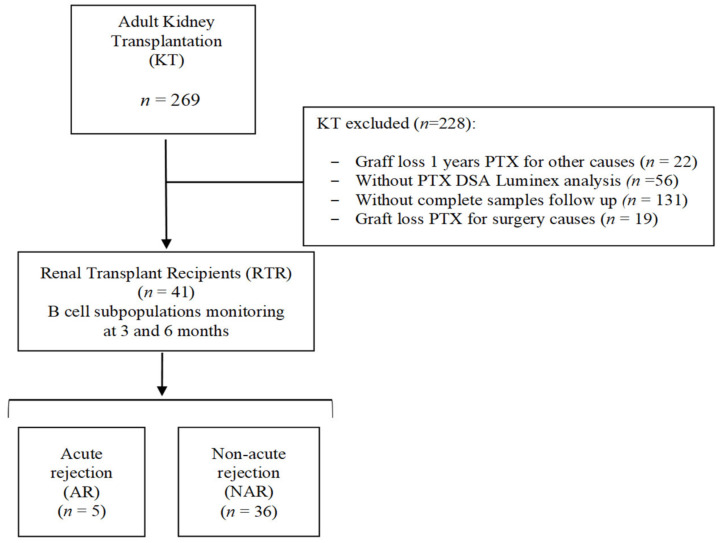
Flow chart of the design and study populations. Abbreviations: AR, Acute rejection; KT, Kidney Transplantation; NAR, Non-acute rejection; PTX, Posttransplant; RTR, Renal Transplant Recipients.

A total of 269 consecutive adult renal transplant patients (KT) were studied and retrospectively analyzed at University Clinic Hospital “Virgen de la Arrixaca” (Spain) (Figure 1). Only those whose kidney transplant was in operation for at least one month after transplantation (PTX) and who had all DSA Luminex determinations for detecting anti-HLA antibodies and all follow-up samples were enrolled. Allograft loss was estimated as a return to dialysis. Thus, the specific B-cell monitoring study was finally performed on 41 renal transplant recipients (RTRs), whose specific clinical and demographic characteristics are shown in Table 2.

The RTRs were classified according to the presence (AR group) or absence (NAR group) of AR during the first year of PTX. Of the 41 total RTRs studied, five (12.2%) experienced acute graft rejection during the first year PTX compared with 36 (87.8%) who maintained stable renal function without rejection during the same period. Of the RTRs with AR, four were classified as acute cellular rejection and one as acute humoral rejection. Five patients in the NAR group had non-DSA anti-HLA antibodies before transplantation, in contrast to the AR group, in which none had performed anti-HLA antibodies. Maintenance therapy consisted of tacrolimus, methylprednisone, and mycophenolic acid. In addition, 19 KTRs (46.3%) received induction therapy (12 Thymoglobulin-Tim and 5 Basiliximab-Bas), with no significant differences between the NAR and AR groups. Within the NAR group, 12 KTRs (33.3%) suffered delayed graft function compared with one in the AR group (20%). No significant differences were observed in age, gender, HLA incompatibilities, and donor type.

### 2.2. Immunosuppressive Treatment

All included recipients received similar triple immunosuppressive therapy, consisting of oral tacrolimus (Prograf, Astellas, Ireland), mycophenolatemofetil (MMF; CellCept, Roche, Switzerland), and prednisolone (Dacortin, Merck, Spain). The tacrolimus (FK) based protocol was started at a dose of (0.10–0.15 mg/kg/day) and the dose was adjusted to maintain a trough level of F.K. in whole blood between 8 and 12 ng/mL during the first-month after-surgery, between 7 and 10 ng/mL at 2–3 months after transplantation and between 5 and 8 ng/mL thereafter. MMF was started at a dose of 2000 mg/day, decreasing to 1000–1500 mg/day during the first month PTX, depending on the white blood cell count.

Methylprednisolone was administered intravenously in doses of 500, 250, and 125 mg/day at transplantation, on days 1–2, and on days 3–4 after surgery. Oral prednisolone was started on the fifth day after surgery at the dose of 20 mg, and then tapered to 5–10 mg/day within 2–3 months of PTX. Some cases were treated with induction therapy based on thymoglobulin or basiliximab, depending on the immune risk before transplantation.

### 2.3. Kidney Rejection Diagnosis

Protocol biopsies were not classically performed in our clinical hospital. The indication for biopsy was the increase in creatinine levels and/or the presence of DSA antibodies on routine evaluation. Acute cellular rejection (ACR) was defined as an increase in serum creatinine of at least 20% above baseline serum creatinine and biopsy-proven rejection (specimens were evaluated by light microscopy and immunofluorescence staining with a marker of classical complement activation (C4d) and classified according to the Banff classification updated in 2017. The diagnosis of acute antibody-mediated rejection (AMR) requires the presence of distinguishable histopathological findings, positive C4d staining in peritubular capillaries, and the concomitant presence of DSA [9]. For the kidney, a consensus was reached that the diagnosis of AMR requires the simultaneous presence of DSA, distinguishable histopathological findings, and deposition of C4d in peritubular capillaries.

Mild acute cellular rejection (Banff grade I) was treated with pulse steroids (500 mg methylprednisolone bolus) and increased maintenance immunosuppression. All other ACR were treated with anti-thymocyteglobulin (ATG) treatment.

AR episodes were further classified as steroid-sensitive rejections (ACR Banff grade I) or steroid-insensitive rejections (ACR Banff grades II and III) and AMR.

AMR was also treated with pulsed steroids and intravenous immunoglobulin (0.25 gr/kg and the last session 1 gr/kg, (maximum 140 g) divided into two doses in combination with plasmapheresis (3 sessions per day, every five days). Later, we administered 500 mg of anti-CD20 (Rituximab, Roche pharmaceuticals) intravenously. Two patients receiving the anti-proteasome inhibitor Bortezomib (Velcade^®^, formerly PS-341) also received anti-AMR treatment.

### 2.4. Monitoring of Subpopulations of B Cells by Flow Cytometry

Monitoring of BL subpopulations was performed by flow cytometryin peripheral blood samples collected before and three and six months after PTX. Pretransplant samples were collected at the transplant center and processed within 24 h. T cells were also monitored, similar to our previous articles [11].

Peripheral blood samples were labeled with the monoclonal antibodies listed in Table 3, using the standard flow cytometry labeling technique.

Briefly, 50 µL of peripheral blood was incubated with 5 µL of each monoclonal antibody for 10 min in the dark. Samples were then lysed with 3 mL of BD FACS Lysing Solution (Becton Dickinson B.D., Bioscience, San Jose, CA, USA) and incubated for 7 min at room temperature. After lysis, samples were centrifuged at 1800 revolutions per minute (rpm) for 5 min and washed with PBS (Phosphate Buffered Saline), as previously published [11,12].

After the labeling procedure, samples were acquired on a FACS Canto II flow cytometer (Becton Dickinson B.D., San Jose, CA, USA). Analysis of the results was performed in the FACS. Diva software (Becton Dickinson B.D., San Jose, CA, USA) evaluated. The absolute number of the different cell subpopulations was determined by applying the relative abundances obtained by flow cytometry to the absolute lymphocyte count obtained in a Medtronic cell counter M16 (Boule Medical, Stockholm, Sweden).

The phenotype of the lymphocyte subpopulations and the visual analysis strategies are shown in Table 4 and Figures S3 and S4. The phenotype of the T and B lymphocyte subpopulations are based on panels from a consortium of studies [13].

### 2.5. Statistical Analysis

Results were expressed as mean ±standard error of the mean (S.E.M.) for quantitative data or as percentages for categorical data. The χ^2^ test or Fisher’s exact test was used for comparison of categorical variables. The verification of data was performed using the Kolmogorov–Smirnov test. For comparison of two groups with variables that do not adjust to normality, Mann–Whitney U test was used. For the comparison of three or more groups, the Kruskal–Wallis test and Dunn’s post hoc test with Bonferroni correction for multiple comparisons were used, and the correlation analyzes were performed using the Spearman index, as previously published [9].

For longitudinal comparison of two related groups, the non-parametric Wilcoxon test for related samples was used. Friedman test with Wilcoxon post hoc was used [11,14].

Evaluation of sensitivity and specificity of a biomarker was performed by generating ROC curves. Discriminatory ability was evaluated by measuring the Area Under Curve (AUC). The Youden index was used to obtain the optimal cut-off value that maximizes sensitivity and specificity [11,14]. To correct the *p*-value multiple comparisons, the Benjamini–Hochberg or Bonferroni method was used. Values of *p* < 0.05 were considered statistically significant in all cases.

The graphs and statistical analyzes were performed in the software Statistical Package for the Social Sciences (SPSS, version 22, Chicago, IL, USA) and GraphPad Prism (version 6, San Diego, CA, USA) and in the programming language R, using for this last R Studio Integrated Development Environment version 3.4.

Before cluster analysis, the relative frequencies obtained for each subpopulation were normalized. Then, the data obtained from the B subpopulations analyzed by flow cytometry were represented/as a matrix, where the columns represent the subpopulations and the rows representing the samples analyzed. To group kidney recipients based on their immunological profiles, Ward’s method was used as the grouping method, using Euclidean distance to measure the degree of similarity between patients. Then, the creatinine levels estimated glomerular filtration rate and frequency of AR of the patients assigned to each cluster were compared using the U-Mann–Whitney test and Fisher’s test, respectively, as previously published [11].

## 3. Results

### 3.1. Monitoring of B Cells Subpopulations in Kidney Recipients

Figure S1 shows the values of subpopulations expressed in relative frequencies and in absolute values of BL subpopulations detected by flow cytometry in peripheral blood samples collected before transplantation and after three and six months PTX.

First, we investigated whether there were significant changes in the levels of the different BL subpopulations during PTX with respect to the baseline values before transplantation.

In terms of relative frequencies, we observe that after six months of PTX, there is a statistically significant decrease from BL to the total number of lymphocytes (Figure S1A; 7.97 ± 0.73% vs. 6.48 ± 0.68%; *p* = 0.049). Within the BL, we observe a significant decrease in BL naive after three (Figure S1B; 68.48 ± 2.50% vs. 62.37 ± 2.03%; *p* = 0.013) and six months of PTX (Figure S1B; 68.48 ± 2.50% vs. 62.65 ± 2.31%; *p* = 0.007). A significant decrease in transitional BL is observed during the period, although only at six months was significant (Figure S1H; 1.89 ± 0.65% vs. 0.71 ± 0.23%; *p* = 0.003). Regarding memory phenotypes (No Class-Switched, Class-Switched, and marginal zone), slight increases were observed during PTX in all of them, but only in the case of the No Class-Switched subpopulation significance was reached after three months PTX (Figure S1E; 8.44 ± 0.99% vs.10.77 ± 0.94%; *p* = 0.037).

In absolute terms, there is a clear trend towards a decrease in total BL during PTX, although the differences with respect to the time before transplantation were not significant. With respect to the BL subpopulations, decrease in the absolute levels of naive BL (Figure S1B; 105.4 ± 17.6 vs. 71.3 ± 9.2 cells/µL; *p* = 0.035) and transitional BL (Figure S1H; 2.79 ± 1.31 vs. 0.59 ± 0.19 cells/µL; *p* < 0.001) was observed after six months of PTX. In memory phenotype B populations, absolute levels remain relatively stable during the PTX period and no significant differences were observed.

### 3.2. Immunological Profile of B Cells in Renal Transplant Patients with Acute Rejection

Two different statistical approaches were used to assess the association of BL with AR and graft function during the first year. First, we used a traditional approach in which the BL subpopulations were evaluated independently and compared separately between the AR and NAR groups. Second, we used a novel approach in which all BL subpopulations were studied together to establish a phenotypic profile and renal transplant recipients (RTRs) were grouped on this basis by cluster analysis to later assess the association of these clusters with the incidence of AR and allograft function.

### 3.3. Association of B-Cells Subpopulations with the Development of Acute Rejection

Comparing the relative frequencies of B subpopulations in the NAR group with the AR group, we find that after six months of PTX, the proportion of BL within total lymphocytes significantly decreased in the AR group (Figure S2A; 6.92 ± 0.76% vs. 3.67 ± 0.53%; *p* = 0.041). The absolute BL values decreased in both groups during the PTX period, but there were no significant differences.

Regarding the BL of the marginal zone, a decrease in relative frequencies was observed under PTX. In absolute terms, a decrease in this subpopulation was also observed in the AR group, but the differences were not significant. No significant differences were observed between the NAR and AR groups for the other BL subpopulations studied.

### 3.4. Association of B Cells with a Kidney Function of the Graft

**Figure 2:**
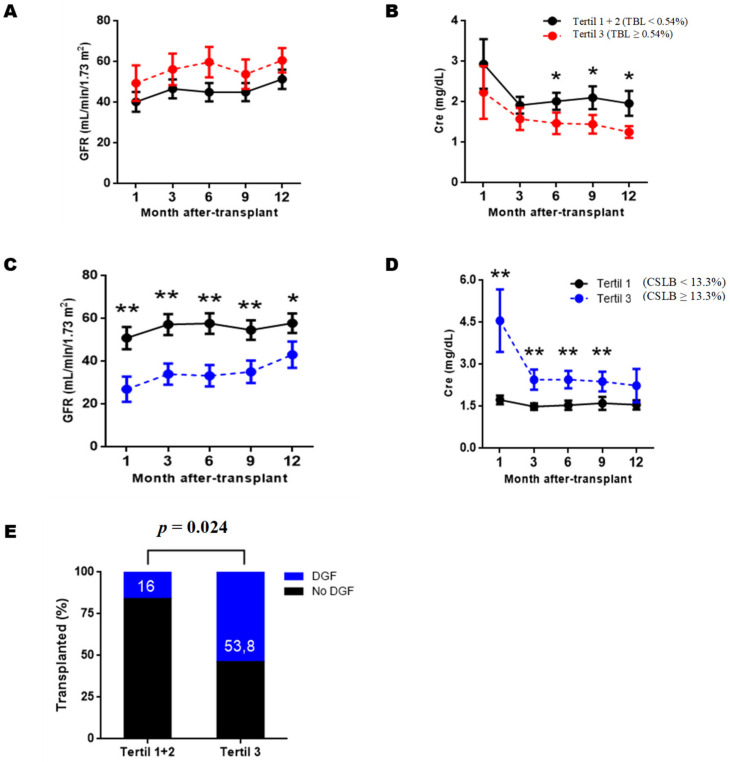
Estimated glomerular filtration and serum creatinine levels of RTRs according to the transitional and Class-Switched B lymphocyte levels at 12 months post-transplantation. Renal recipients were grouped into tertiles based on transitional B lymphocytes’ relative frequencies (TBL) and Class-Switched (BLCS). At each post-transplant time, the estimated glomerular filtration rate (GFR) (**A**,**C**) and serum creatinine (Cre) (**B**,**D**) of the two groups were compared using the U-Mann–Whitney test. Fisher’s exact test (**E**) was used to compare delayed graft function incidence (DGF). Values are represented as the mean ± SEM. Values of *p* < 0.05 were considered statistically significant. * *p* < 0.05. ** *p* < 0.01.

BL subpopulations with graft function, we divided the RTRs into three groups based on the tertiles obtained for each subpopulation. In this way, the RTRs assigned to tertile 1 would be those with the lowest values for a given subpopulation, those assigned to tertile 2 would be in the middle range, and those assigned to the tertile 3 would be in the higher levels. We then compared the estimated glomerular filtration rate and serum creatinine obtained during the first year of PTX between tertiles. The results show that after three months of PTX, BL transitional (BLT) and Class-Switched BL (CSBL) were correlated with serum creatinine levels and graft function (Figure 2).

RTRs assigned to tertile 3 according to BLT values (BLT ≥ 0.54%) have significantly lower serum creatinine levels than the RTRs tertiles 1 and 2 at six (*p* = 0.030), nine (*p* = 0.026) and twelve months (*p* = 0.023) (Figure 2B). Although a slight trend was observed at three months, it did not become significant (Figure 2A, *p* = 0.091). However, when comparing the estimated glomerular filtration levels, no PTX significant differences were observed at any time point.

RTRs according to CSBL levels, we find that those assigned to tertile 3 (CSBL ≥13.3%) had significantly lower renal function throughout the first year PTX than those RTRs assigned to tertiles 1 and 2 (CSBL < 13.3%). Regarding estimated glomerular filtration rate, we observed significant differences at one month (*p* = 0.005), three months (*p* = 0.003), six months (*p* = 0.001), nine months (*p* = 0.005) and twelve months of PTX (*p* = 0.047) (Figure 2C). Regarding serum creatinine levels, significantly higher values were observed in tertile three at one month (*p* = 0.009), three months (*p* = 0.004), six months (*p* = 0.003), and nine months PTX (*p* = 0.004) (Figure 2D).

Because significant differences were observed from the first month of PTX, we wondered whether CSBL levels might reflect clinical events during the first days of PTX other than rejection. To this end, we compared the incidence of infections, delayed graft function (DGF), or use of induction therapies between tertiles 3 and tertiles 1 + 2. Figure 2E shows that the incidence DGF, defined as the need for dialysis during the first week of PTX was significantly higher in recipients assigned to tertile 3 (*p* = 0.024). Regarding induction therapies, the proportion of RTR from tertile 3 who was administered thymoglobulin or basiliximab was higher than in tertiles 1 and 2, but the differences were not significant (66.6% vs.45%; *p* = 0.291).

### 3.5. Analysis of the Clusters of B Subpopulations

Prior to cluster analysis, the relative frequencies of each subpopulation were normalized. The values obtained from the normalization (Z score) were used to perform a cluster analysis, which we used to group the RTRs based on the phenotypic profile of BL. Next, we compare the incidence of AR and graft function during the first year PTX between the previous analysis clusters.

**Figure 3:**
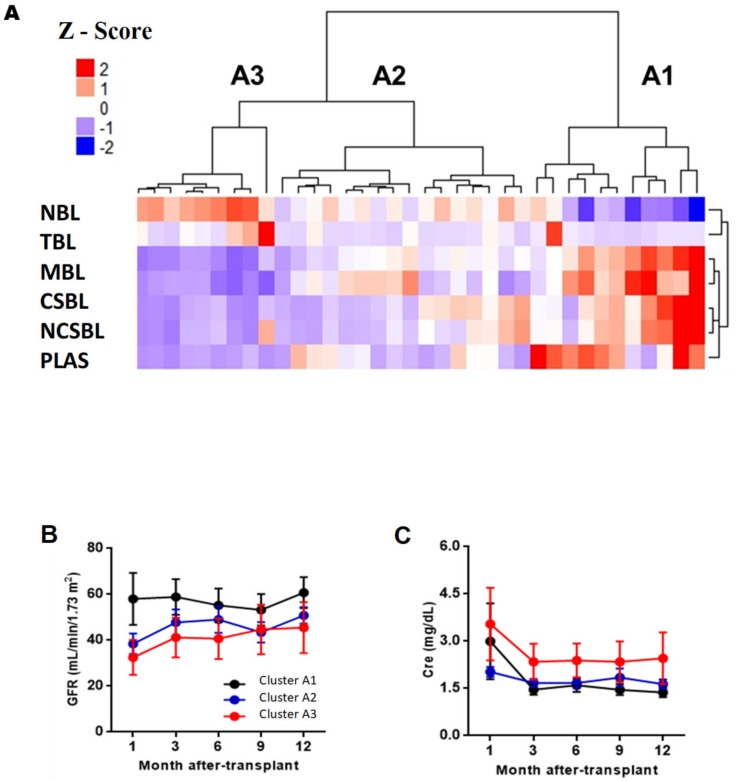
Analysis of BL clusters at pre-transplantation and evolution of the estimated glomerular filtration rate and Cre. (**A**) Heatmap, where they represent a matrix in which the rows are the BL subpopulations and the columns the RTRs. The color grading represents the normalized values (Z-Score) obtained for each subpopulation. Evolution of the estimated glomerular filtration levels (GFR) (**B**) and serum creatinine (Cre) (**C**) during the first year after transplantation of each cluster. The FG and Cre values at each time were compared between the clusters using the Kruskal–Wallis test and Dunn’s posthoc test with Bonferroni correction for multiple comparisons. *p* < 0.05 were statistically significant. Abbreviations: NBL, Naive B Lymphocytes; TBL, Transitional B Lymphocytes; MBL, Memory B Lymphocytes, CSBL, Class-Switched B Lymphocytes; PLAS, Plasmablasts; NCSBL, No Class-Switched B Lymphocytes, ZBL, Zone B Lymphocytes.

Figure 3A shows that three major clusters were obtained at pre-transplantation (clusters A1, A2, and A3). Cluster A1 was characterized by having the highest ratio of memory to naïve B lymphocytes compared, with the other clusters, with a high proportion of memory B lymphocytes (33.7 ± 12.5%) and a low proportions of naive BL (52.6 ± 13.9%). It also had high levels of plasmablasts (3.02 ± 1.47%) and LBT (1.42 ± 2.53%). Cluster A3 had an opposite phenotype to A1, with a decreased ratio of memory/naïve, a low proportion of memory BL (7.76 ± 3.15%), and a high proportion of naive BL (84.6 ± 6.74%). It had a low proportion of plasmablasts (0.27 ± 0.17%) and a high proportion of LBT (4.29 ± 7.06%). Cluster A2 had an intermediate phenotype between clusters A1 and A3, with an intermediate memory/naive ratio, with a frequency of 70.2 ± 5.62% of naive BL and 18.3 ± 3.85% of memory BL The frequencies of plasmablasts and LBT of cluster A2 were 1.12 ± 0.74%, and 0.85 ± 0.74%, respectively. No significant differences were observed when comparing AR and graft function frequency between A1, A2, and A3 (Figure 3B,C; Table S1).

**Figure 4:**
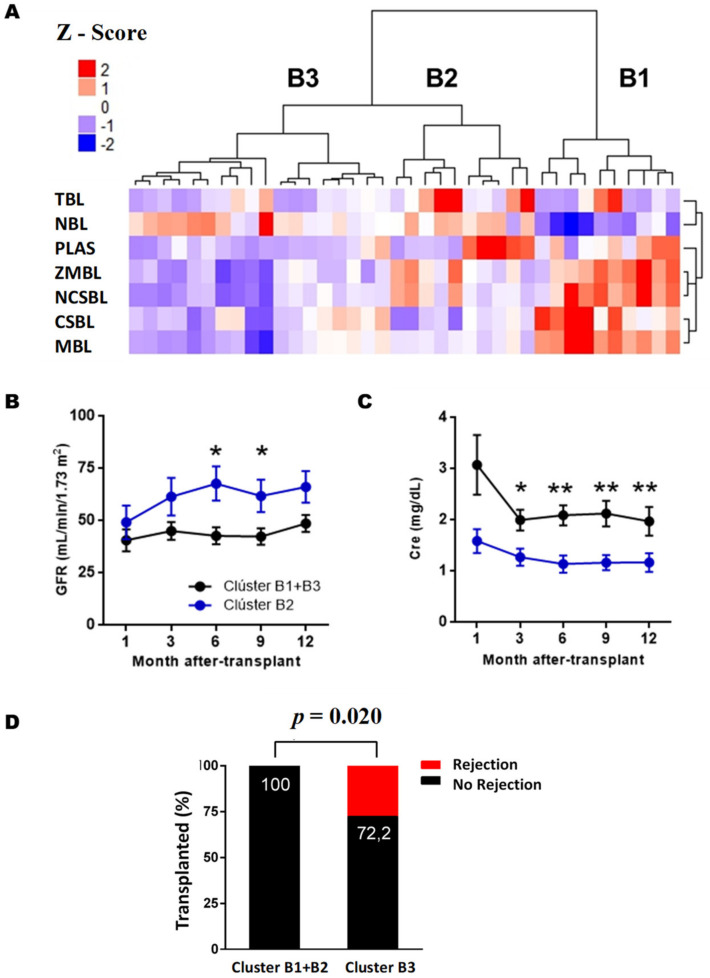
Analysis of the BL clusters at three months post-transplantation. (**A**) Heatmap represents a matrix in which the rows are the BL subpopulations and the columns the RTRs. The color grading represents the normalized values (Z-Score) obtained for each subpopulation. Evolution of the estimated glomerular filtration levels (FG) (**B**) and serum creatinine (Cre) (**C**) during the first year after transplantation of each cluster. In figure (**D**), the numbers on the bars’ black background indicate the percentage of RTRs free of rejection of each group. Data are represented as the mean ± SEM. Statistical analyses were performed using the Kruskal–Wallis test and Dunn’s posthoc test with Bonferroni correction for multiple comparisons. Values *p* < 0.05 were considered statistically significant. Abbreviations: NBL, Naive B Lymphocytes; TBL, Transitional B Lymphocytes; MBL, Memory B Lymphocytes, CSBL, Class-Switched B Lymphocytes; PLAS, Plasmablasts; NCSBL, No Class-Switched B Lymphocytes, ZBL, Zone B Lymphocytes. * *p* < 0.05. ** *p* < 0.01.

After three months of PTX (Figure 4A), cluster B1 was characterized by a high BL memory/naive ratio, cluster B2 by an intermediate ratio, while cluster B3 showed a high-grade phenotype of LB naive. The number of plasmablasts was decreased in cluster B3 compared to clusters B1 and B2. As for LBTs, cluster B2 was the one with the highest values. Within the memory phenotype, cluster B1 had increased levels of both CSBL and BL No Class-Switched (LBNCS). Between clusters B2 and B3, both had similar levels of CSBL, but cluster B2 had a higher number of LBNCS. As for the BLs of the marginal zone, cluster B3 had lower values compared to the other clusters.

In the association between the clusters obtained after three months of PTX and renal function, we observed that cluster B2 had significantly lower serum creatinine levels (Figure 4C) and higher estimated glomerular filtration rate (Figure 4B) than the RTRs assigned to clusters B1 and B3 (Table S2).

Regarding AR incidence, patients in cluster B3 had a higher incidence during the first year PTX than clusters B1 and B2 (*p* = 0.020). Further, 27.3% of cluster B3 (5/18) suffered AR during the first year PTX, while none of the clusters B1 and B2 had an AR.

Finally, to check whether a clinical variable could influence our results, clusters B1, B2 and B3 were compared with the incidence of DGF, which according to the literature, is a risk factor for rejection and worse PTX renal function. However, there were no significant differences in these variables between the two groups when induction therapies were used, which could have an impact.

Based on these results, we can conclude that cluster B2 has a protective phenotype against AR and adequate renal function, characterized by high LBT and plasmablasts levels. Cluster B1, with a high memory/naive ratio and intermediate LBT level, showed an intermediate clinical prognosis, with a low risk of rejection but worse renal function than cluster B2. At the same time, cluster B3 had the worst prognosis with low LBT and plasmablasts levels, with a high risk of rejection and poor renal function.

**Figure 5:**
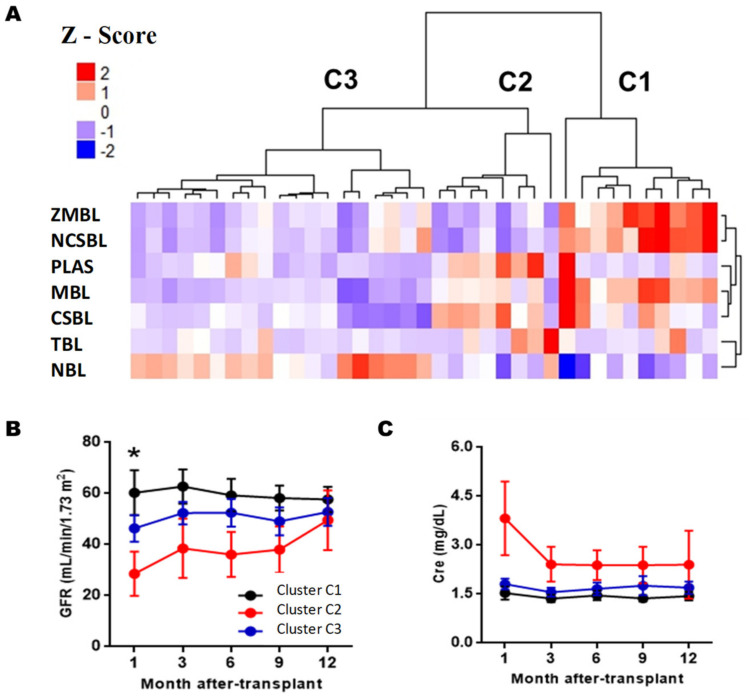
Analysis of BL clusters at six months post-transplantation. (**A**) Heatmap represents a matrix in which the rows are the BL subpopulations and the columns the RTRs. The color grading represents the normalized values (Z-Score) obtained for each subpopulation. Evolution of the estimated glomerular filtration levels (GFR) (**B**) and serum creatinine (Cre) (**C**) during the first year after transplantation of each cluster. The FG and Cre values at each time were compared between the clusters using the Kruskal–Wallis test and Dunn’s posthoc test with Bonferroni correction for multiple comparisons. Values of *p* < 0.05 were considered statistically significant. Abbreviations: NBL, Naive B Lymphocytes; TLB, Transitional B Lymphocytes; MBL, Memory B Lymphocytes, CSBL, Class-Switched B Lymphocytes; NCSBL, Non-Class-Switched B Lymphocytes, MZBL, Marginal Zone B Lymphocytes; PLAS, Plasmablasts. GF, glomerular filtration. * *p* < 0.05 between clusters C1 and C2.

On the other hand, Figure 5A shows that three large clusters are obtained six months PTX (clusters C1, C2, andC3). Clusters C1 was characterized by having the highest memory/naïve ratio, with equal BL memory levels (36.6 ± 10.1%) and LB naive (48.9 ± 13.0%). It had mean values of LBT (0.56 ± 0.72%) and plasmablasts (1.93 ± 2.70%). Cluster C2 was characterized by a lower ratio of memory/naive than cluster C1, with LB naive levels of 57.4 ± 9.36% and LB memory of 23.3 ± 4.87%. It had high levels of LBT (2.76 ± 1.46%) and plasmablasts (1.56 ± 2.47%) and was the cluster with the highest levels of CSBL (16.1 ± 5.06%). Cluster C3 had the lowest memory/naive ratio, with 72.0 ± 8.13% naive BL and low levels of LBT and plasmablasts. When comparing the incidence of AR and graft function, differences between clusters C1 and C2 were only observed in glomerular filtration rate estimated after one month of PTX (*p* = 0.025), but at later times the differences disappeared (Table S3).

### 3.6. Cluster B2 Patients Have Increased Levels of HLA-DR+ Regulatory T (Treg) and Activated T Cells

Following the results of the cluster analysis, we explored whether there were also differences in the major subpopulations of T lymphocytes in the RTRs assigned to cluster B2. Using flow cytometry, we analyzed the naïve CD4 and CD8 memory and activated HLA-DR+ populations, as well as the levels of Tregs, as previously published [13].

**Figure 6:**
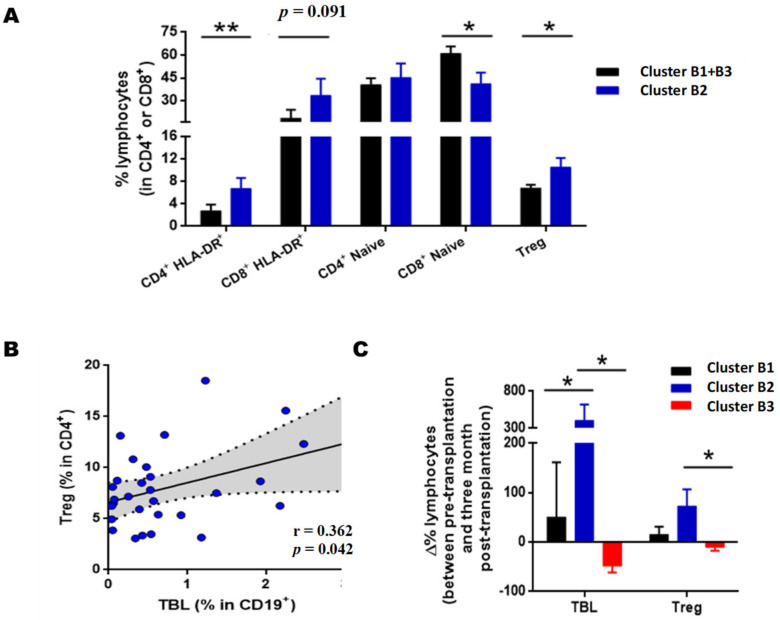
T cell phenotype of clusters B1, B2, and B3. (**A**) Relative frequencies of activated HLA-DR+, naive and regulatory T lymphocytes (Tregs) at three months post-transplantation of clusters B1 + B3 and B2. Comparisons made using the Mann–Whitney U test. (**B**) Correlation between levels of Treg and transitional B lymphocytes (TBL). Analysis performed using the Spearman index. The r corresponds to the correlation coefficient, and the shaded area indicates the 95% confidence interval. (**C**) Percentage variation of the levels of TBL and Treg between pre-transplantation and three months after-transplant. Comparisons made using the Kruskal–Wallis test and Dunn’s post hoc test with Bonferroni correction for multiple comparisons. Values expressed as the mean ± SEM. *p* values < 0.05 were considered statistically significant for all analyses. * *p* < 0.05; ** *p* < 0.01.

The data show that the cluster B2 recipients had increased levels of activated CD4 HLA-DR+ (*p* = 0.005) and activated CD8HLA-DR+, after three months of PTX, although this was not statistically significant in the latter (*p* = 0.091) (Figure 6A). On the other hand, recipients in cluster B2 had a lower number of naive CD8 (CD8+CD45RO-) than recipients assigned to clusters B1 and B3 (Figure 6A, *p* = 0.023). A significant increase was observed in Tregs lymphocytes after three months of PTX (*p* = 0.038), which correlate positively with transitional B lymphocyte levels (Figure 6B, r = 0.362, *p* = 0.042). When examining the variations in the percentage of transitional B lymphocytes and Tregs after three months of PTX for pre-transplant baseline values (Figure 6C). Our data show that the recipients of cluster B2, with a good clinical prognosis, were the recipients with the highest LBT and Treg increase. The increase in LBT levels in cluster B2 was significant with respect toclusterB1 (*p* = 0.034) and B3 (*p* = 0.018). Regarding Tregs, the difference between pre-and post-transplantation was also significant when comparing clusters B1 and B2 (*p* = 0.015).

Therefore, cluster B2 recipients with a good prognosis had a large increase in LBT and Tregs during the first months of PTX. The RTRs assigned to cluster B1, with an intermediate prognosis, also showed an increase in LBT and Tregs, but this increase was less pronounced in cluster B2. Finally, the RTRs of cluster B3, with a poor prognosis, showed a decrease in LBT and Tregs values.

### 3.7. Effect of Thymoglobulin on B Cell Subpopulations

Given the relevant role attributed to transitional BLs in the clinical status of RTRs, we decided to explore whether thymoglobulin, a drug commonly used as induction therapy, could affect the absolute and relative levels of this subpopulation during the PTX period. To do this, we compared the change in transition BL to the pre-transplantat period in RTR administered thymoglobulin (*n* = 12) and a group without induction therapy (*n* = 19). To avoid possible confounding factors that could influence the results, we included only RTR without AR.

**Figure 7:**
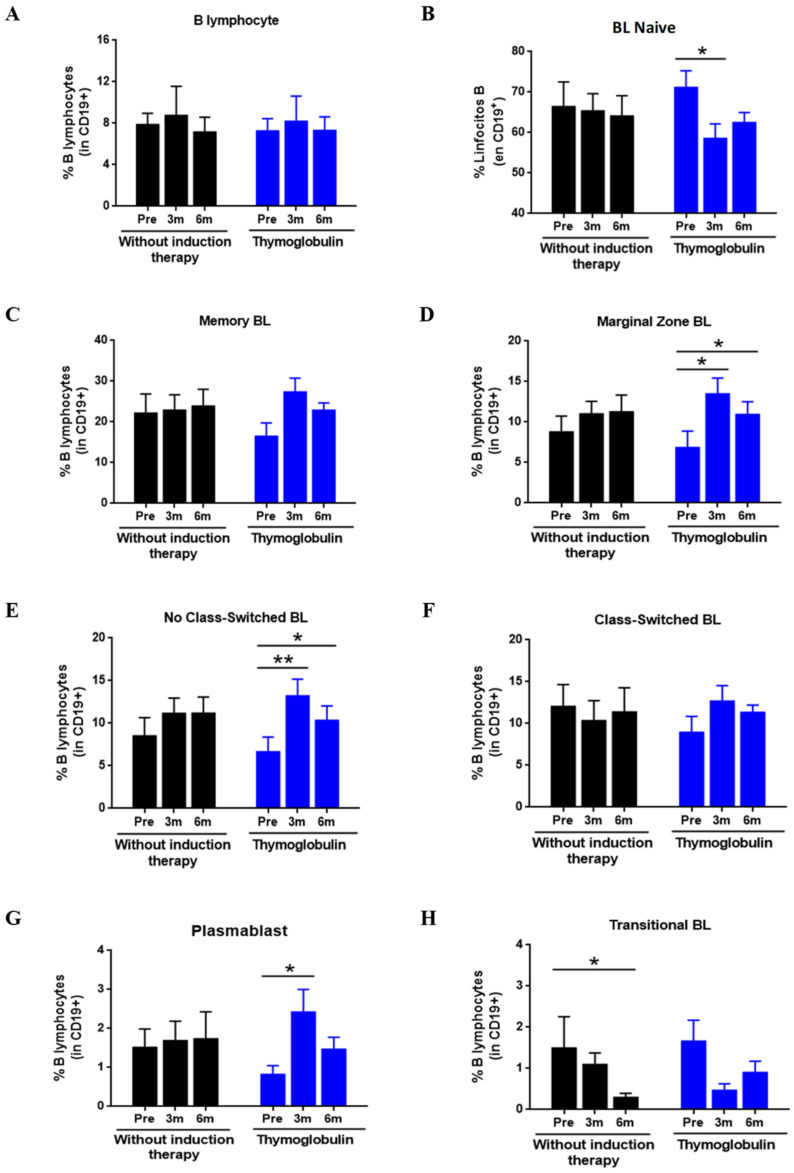
Effect of thymoglobulin on B lymphocyte subpopulations in pre- and post-transplant. The figure shows the relative frequencies of the BL subpopulations in RTR administered thymoglobulin (Blue bars, *n* = 12) and did not receive induction therapy (Black bars, *n* = 19). The values show mean ± SEM. The comparison within each group between pre-transplant and post-transplant values was performed using the Wilcoxon test for related samples. The comparison at each time between the group without induction and the thymoglobulin group was carried out using the Mann–Whitney U test. Values of *p* < 0.05 were considered statistically significant. * *p* < 0.05; ** *p* < 0.01. Abbreviations: NBL, Naive B Lymphocytes; TLB, Transitional B Lymphocytes; MBL, Memory B Lymphocytes, CSBL, Class-Switched B Lymphocytes; NCSBL, Non-Class-Switched B Lymphocytes, MZBL, Marginal Zone B Lymphocytes; PLAS, Plasmablasts. GF, glomerular filtration.

Figure 7 shows that the relative frequencies of BL of RTRs treated with thymoglobulin were altered at PTX compared to pre-transplant values. Initially, the total B cell frequency was not significant (Figure 7A). However, the relative frequency of naive BL (Figure 7B) showed a decrease after three months of PTX (71.2 ± 12.6% vs.58.6 ± 11.7%, *p* = 0.038) and after six months of PTX (71.2 ± 12.6% vs.62.5.6 ± 7.7%, *p* = 0.086), although it was not significant in the latter case. The decrease in naive BL was also contrasted with an increase in BL in the marginal zone (Figure 7D) both at three months (6.9 ± 6.2% vs. 13.5 ± 6.2%, *p* = 0.015) and at six months PTX (6.9 ± 6.2% vs. 10.9 ± 5.1%, *p* = 0.038). No Class-Switched Lymphocytes (Figure 7E) also increased in the thymoglobulin-treated both three (6.7 ± 5.4% vs. 13.2 ± 6.3%, *p* = 0.008) and six months PTX (6.7 ± 5.4% vs. 10.3 ± 5.5%, *p* = 0.011). Regarding plasmablasts (Figure 7G), an increase was also observed at three (0.83 ± 0.6% vs. 2.43 ± 1.9%, *p* = 0.028) and six months of PTX (0.83 ± 0.6% vs. 1.47 ± 0.9%, *p* = 0.066), although it was not significant after six months. No significant differences were observed in BL memory, Class-Switched, and transition scores in the traditional or classical approach.

In the RTR group without induction therapy, no significant differences were observed in any BL subpopulation except for transitional BLs (Figure 7H), where a significant decrease was observed after six months of PTX compared to pre-transplant (1.51 ± 2.3% vs. 0.31 ± 0.3%, *p* = 0.033). Next, we compared the BL levels between both groups at each PTX time point, but we found no significant differences for any subpopulation.

In terms of absolute levels (data not shown), we found no significant differences in the thymoglobulin treated group. In the group without induction therapy, we observed that six months of PTX, resulted in a significant decrease in transitional BL (1.61 ± 1.7 cells/µL vs.0.34 ± 0.4 cells/µL, *p* = 0.003).

In our study, no differences were also observed when comparing at any time point between the groups with thymoglobulin and without induction therapy.

## 4. Discussion

B lymphocytes have gained importance in the field of renal transplantation in recent years [2,15,16]. The discovery of regulatory BLs (Bregs), a B subpopulation with immunosuppressive capabilities, has prompted the scientific community to rethink the role of BLs in allospecific responses. BLs are no longer considered as mere producers of antibodies, but have gained great importance in transplantation due to their newly discovered immunoregulatory capabilities [15]. Although several studies have been published on the dynamics of BL in renal transplantation, data on their impact on AR and graft function are still minimal [16].

In the present work, we used flow cytometry to monitor the major subpopulations of BL in kidney transplant patients in samples collected before and after transplantation. To establish the relationship between BL subpopulations, the incidence of AR, and graft function, we used two methodological approaches, traditional statistical analysis, and cluster analysis. Our results show that, after three months of PTX, patients with a B phenotype enriched with transitional BL and plasmablasts have a better renal function and a low incidence of AR. We also studied the effect of thymoglobulin, one of the drugs most commonly used as induction therapy, on B subpopulations, and showed that this drug causes a decrease in the relative frequency of naive BLs.

Primarily, we study the PTX evolution of BLs. Our results show that the absolute values and relative frequencies of BL exhibit decreasing PTX periods. Although some studies are consistent with our findings [17], others [18] observed an increase in BL at one week PTX, and recovery of pre-transplant levels at three months PTX. All patients included in this study [19] received induction therapies, so the rapid recovery of basal levels of BL may be associated with homeostatic lymphoproliferative processes after lymphocyte depletion. Examining the BL subpopulations, we see that the decline of BL is mainly due to the naive and transitional subpopulations, while the BL with memory phenotype remains relatively stable in the PTX period. Schlößer et al. [17] also observed the maintenance of BL memory levels throughout the first year PTX. Other studies also reported a decrease in immature phenotypes without affecting the mature/memory phenotypes. Chung et al. [12] also showed in in vitro cultures that tacrolimus and mycophenolatemofetil significantly reduced immature LB levels, while mature and memory phenotypes were not affected, which may explain the effect observed in our cohort.

When analyzing the relationship between AR and the BL subpopulations, we observed a clear tendency in RTR with AR, relative memory BL levels lower than those of the NAR group. However, the differences did not reach statistical significance. These data confirm the observations of Zarkhin et al. [20], in whose study the decrease in naive/memory ratio was associated with AR. Instead, Newel et al. [8] showed that tolerant RTRs had a higher frequency of naive and transitional BL and a lower number of memory B lymphocytes than non-tolerant RTRs. On the other hand, several studies show the association between transient BL and transplant tolerance and AR prevention [21,22].

Transitional BL is a population commonly studied in kidney transplantation because it is enriched in Bregs with immunosuppressive capacities. However, in our study, we did not observe any differences between the NAR and AR groups in the study of transitional BL. In our study, we did not examine the expression of IL-10 within the BL transitions, which may explain the absence of differences between groups. However, there is still controversy about the best way to analyze transitional BLs for their use as a biomarker. While Svachova et al. [23] show the usefulness of analyzing the total transitional BL to determine the risk of AR, other authors, such as Cherukuri et al. [2], indicate that the analysis of IL-10 expression is necessary in this subpopulation. In addition, the low incidence of rejection in our cohort during the surveillance and follow-up period is a critical limiting factor. Therefore, future studies with longer cohorts will be necessary to confirm the results obtained.

We grouped RTRs based on the relative tertile frequencies of each BLs subpopulation to examine the relationship between BLs subpopulations and graft function. Our results show that the RTRs with high levels tertile 3 of transitional and Class-Switched BL showed a relationship with renal function. Kidney graft rejecting recipients (TKRs) with high transitional BL reflected significantly lower creatinine levels from six months PTX onwards, although no statistical differences in estimated glomerular filtration rate were observed. Along these lines, the association between transitional BL levels and renal function has already been reported previously [10] and transitional BL with graft dysfunction. Thus, RTR with high levels of T1-type transitional BLs, with a greater capacity for IL-10 expression, exhibited better PTX evolution [23].

Regarding the Class-Switched BLs, we observed that the RTRs with high levels of this subpopulation had worse renal function already in the first month of PTX. The differences found in the early stages of transplantation led us to wonder if any clinical event could be behind these abnormally high BL Class-Switched levels. Elevated memory levels BL are associated with allospecific reactions and other clinical contexts, such as CMV viremia.

After evaluating different clinical scenarios—infections, medications, and delayed graft function (DGF), we found that BLs. Class-Switched tertil 3 patients had a higher incidence of DGF and a higher proportion of TKR treated with induction therapies. Patients with DGF tended to have lower renal function levels and a higher rejection rate [24]. The decrease in naive phenotype due to pharmacological causes would explain the accumulation of BLs. Class-Switched in this group. In this sense, Svachova et al. [23] already observed how in the THRs with thymoglobulin induction, the proportion of BLs. Class-Switched one week of PTX increased. On the other hand, dialysis patients have defects in B differentiation leading o marked B lymphopenia [24], and it is likely that in addition to the effects of induction therapies, the additional dialysis sessions, that patients must undergo negatively affect the reconstitution of naive BL during the first months of PTX in patients with RFI/DGF. However, there are few studies on the effect of DGF in B subpopulations, so our hypotheses need to be confirmed in subsequent studies and with a larger number of patients.

On the other hand, the immune system is an interconnected network where valuable information can be lost by studying its parts in isolation. Therefore, we decided to use cluster analysis to establish a phenotypic pattern of BL, considering all subpopulations as a whole, in order to establish relationships with the main adverse events of transplantation. This methodology has been used in other melanoma and hematopoietic transplantation studies, including [25,26]. Our data reveal that RTRs can be classified into three clusters after three months of PTX, based on BL and the phenotypic profile of BL, and that these are correlated with the clinical evolution of PTX. TKRs with good PTX evolution, good renal function, and AR absence are characterized by a higher frequency of transitional BL and plasmablasts than those grafts with poorer evolution. Zarkhin et al. [20] observed that the naive/memory ratio was decreased in RTRs with AR. However, we did not obtain these results, as they were similar in both groups with excellent and poor PTX evolution. The discrepancies between our study and this one may be due to the differences between the study cohorts and the time taken to collect the samples. While our cohort includes adult patients, Zarkhin et al. [20] consist entirely of pediatric patients administered rituximab, which depletes BLs. In addition, the sampling of this study occurred at the time of rejection, whereas our study performed e monitoring, so it is likely that the change in naive/memory ratio occurs weeks or days before clinically apparent graft damage. Svachova et al. [23] observed that a decreased transitional BL levels after three months PTX is associated with a greater likelihood of developing AR.

Studies with intolerant patients show that they have increased transitory and naive BL levels and reciprocally decreased with a memory phenotype. However, as we previously argued, this effect may be due to progressive withdrawal of immunosuppression [8,27]. In addition to transitional BL, plasmablasts are another population that differentiates RTRs with good and poor prognosis, with an increased group with good PTX evolution. Plasmablasts have also been reported to exert immunosuppressive functions through the secretion of IL-10. Therefore, the increase of RTRs with a good prognosis could affect transplant tolerance’s subpopulation [28,29]. However, histological findings in rejection specimens also show the relationship of plasmablasts to allospecific humoral responses [26]. Therefore, our data suggest a dual role of plasmablasts, and how to target them to immunosuppressive functions requires further but represents a new therapeutic tool that would favor the induction of graft tolerance. We extended our results of the cluster analysis after three months of PTX, with further analysis evaluating different T subpopulations. The group’s RTRs of the good PTX prognosis group had significantly more CD4+HLA-DR+ cells, naïve CD8 cells and Tregs than those of the low prognosis group. We found the high levels of HLA-DR+ activated T cells striking as they tend to correlate with rejection events and poor prognosis [14,30]; however, this was not the case in our cohort. It was observed that activated HLA-DR+ T cells increase in situations such as infections or inflammatory processes, but we did not observe any differences in our cohort for these reasons [9].

On the other hand, we observed that RTRs with a good prognosis had significantly more Tregs. Moreover, we observed that Tregs levels were positively correlated with transitional BL levels. As we mentioned earlier, transitional BLs are enriched in Bregs. Bregs are able to synthesize numerous cytokines such as IL-10 and TGF-β which are able to induce the differentiation of T cells towards Tregs, which would explain the positive correlation between these subpopulations found in our study [31]. When analyzing the PTX evolution of transitional Tregs and BL for the pre-transplant period, we observed that those RTRs with a good prognosis had the highest increases in these subpopulations. These results suggest that a relative increase in subpopulations with immunosuppressive capacities such as Tregs and transitional BLs in the early stages of transplantation increases graft tolerance and contributes to a better PTX evolution, as shown in numerous studies [30,32,33].

In our study, we also investigated the effect of thymoglobulin on BL subpopulations, as this drug is widely used in renal transplantation. Our data show that RTRs receiving thymoglobulin have a PTX reduction in the frequency of naive BL associated with a relative increase in memory BL levels; however, when comparing BL levels between RTR with and without thymoglobulin, we did not obtain statistically significant differences. Although Zand et al. [11] showed the in vitro anti-B effect of thymoglobulin by activating apoptotic pathways, several studies suggest a small in vivo effect on BL, even at high concentrations [34]. Gurkan et al. [35] observed no differences in the absolute levels of BL of RTR treated with thymoglobulin. However, unlike us, they observed a decrease in the relative frequency of memory BL. This study suggests that depletion of T lymphocytes may disrupt T-B cooperation and alter the proper differentiation of naive BLs to memory BLs. Svachova et al. [23] compared the absolute levels between transplanted patients with thymoglobulin and basiliximab PTX in the group treated with thymoglobulin, confirming the slight effect of thymoglobulin on BL. However, our data confirm the observations made in other studies. The effect on BL was evaluated several months after treatment. Therefore, we do not know what happens in the first days after treatment. This limitation also applies to other studies [34], where the first sample had a month after treatment. It is likely that the effect of thymoglobulin is only observable in the short term and that the subsequent homeostatic lymphoproliferation of BL makes it difficult to detect differences in samples collected several months after drug administration.

Our results support previous observations that increased PTX graft levels BL are associated with a better prognosis, and exert a protective effect against rejection. KTRs, free of rejection and better renal function, show a phenotypic profile of BL subpopulations distinct from those with poorer PTX evolution; therefore, cluster analysis allows the formation of prognostic groups as early as three months of PTX. Moreover, an increase in transitional BL PTX correlates with an increase in Tregs’ levels, confirming that RTR with good evolution has a more significant increase in subpopulations with an immunosuppressive capacity [27].

In any case, this study has important limitations that could affect the observed results, and should be considered in future studies. The limited number of patients monitored, the scarcity of clinical information about the patients could also mean that it is not clear whether the changes in the observed lymphocyte subpopulations could be an effect of time, different clinical events (e.g., infections), or differences in therapeutic options for subclinical rejections. Another limitation is that biopsies, the lymphocyte populations that may infiltrate the graft, may not match those found in the periphery. The number and type of lymphocyte subpopulations present in the peripheral blood may not necessarily match the transplanted organ.

On the other hand, certain patients’ belonging to a group and different cluster groups were carried out. In each case, no statistically significant differences were obtained, or they could not even be calculated because of the small subgroups of patients that appeared, and no conclusion can be drawn, but in any case, they will be considered in future studies.

In conclusion, monitoring BL subpopulations may serve as a non-invasive tool to improve the immunological follow-up of patients after renal transplantation. However, further studies are needed to confirm the obtained results o, define cut-off values, and standardize more optimal and even tailored protocols.

## Figures and Tables

**Table 1:** Main B cells types, functions, and expression biomarkers.

Type of B Cells	Functions	Markers
Transitional B Cells	These cells link immature B cells in the bone marrow and mature B cells in lymphoid organs. These cells have differentiated into B cells from typical myeloid progenitor cells in the bone marrow; however, they are not yet mature.	These are characterized by IgM and IgD expression, by the high expression of CD24 and CD38, and by the absence of the memory marker CD27.
Naïve B Cells	Naïve B cells are located in the secondary lymphoid organs. They are mature but not yet activated. Naive B lymphocytes can differentiate into plasmablasts and plasma or memory B cells in response to stimulation by specific antigens.	These cells have phenotypic markers CD24+CD38+CD27−.
Plasmablasts and Plasma Cells	Plasma cells are long-lived differentiated cells whose function is the production of antibodies. Plasmablasts are also antibody-producing cells, but unlike plasma cells, they are short-lived. Plasma cells migrate to the bone marrow, where they continue to produce antibodies to protect against re-infection.	Phenotypically, plasmablasts and plasma cells are characterized by the absence of CD20 expression and the high expression of CD38.
Memory B Cells	Memory B cells continually recirculate around the periphery and rapidly differentiate into antibody-producing plasmablasts upon interaction with T cells after specific antigen recognition. This memory B response is characterized by being more potent towards antigens than the primary B responses and producing responses with greater affinity and the isotype change of immunoglobulins.	These cells phenotypically express CD20+, CD27+, CD38^−^.

**Table 2:** Demographic data and clinical characteristics of the transplanted patients included in the B lymphocyte monitoring study.

	RTR (*n* = 41) *n* (%)	NAR (*n* = 36) *n* (%)	AR (*n* = 5) *n* (%)	*p* ^a^
Age (years; mean ± SEM)	56.6 ± 2.5	57.7 ± 1.6	51 ± 7.9	0.449
Gender (Male/Female)	(28/13)	(23/13)	5 (5/0)	0.104
IncompatibilitiesHLA ^b^	3.9 ± 0.4	3.8 ± 0.2	4.0 ± 1.1	0.548
Live Donor (%)	2 (4.8)	2 (5.5)	0 (0)	0.589
Preformed anti-HLA antibodies (%)	5 (12.2)	5 (13.8)	0 (0)	0.374
Induction Therapy (Tim/Bas)	19 (14/5)	17 (12/5)	2 (2/0)	1.000
Delayed graft function (%)	13 (29.3)	12 (33.3)	1 (20)	1.000
Type of rejection (Cellular/Humoral)	5 (4/1)	-	5 (4/1)	-

NRA, No Acute Rejection; RA, Acute Rejection; RTR, Renal transplant recipient; SEM, standard error of the mean. Tim, Thymoglobulin; Bas, Basiliximab. Quantitative data expressed as the mean ± standard error of the mean (SEM). ^a^ Comparisons were made using Fisher’s exact test or X^2^ for qualitative variables and using the nonparametric Mann-Whitney U test for quantitative variables. Values of *p* < 0.05 were considered statistically significant. ^b^ Total differences between donor and recipient concerning the HLA-A. HLA-B and HLA-DRB1 genes.

**Table 3:** Antibodies panel to monitoring T and B cells subtypes by flow cytometry.

Fluorocromes	Tube 1: Lymphocytes B		Tube 2: Lymphocytes T	
	Antibody (Clon)	Manufacter	Antibody (Clon)	Manufacter
FITC	CD19 (HIB19)	BD Biosciences	CD19 (HIB19) CD4 (RPA-T4)	BD Biosciences
PE	IgM (G20-127)	BD Biosciences	CD25 (2A3)	BD Biosciences
PE-Cy7	CD27 (M-T271)	BD Biosciences	CD45RO (UCHL1)	BD Biosciences
APC	CD38 (HB7)	BD Biosciences	-	BD Biosciences
APC-Cy7	CD24 (ML5)	BD Biosciences	HLA-DR (L243)	BD Biosciences
PerCP	CD45 (HI30)	BioLegend	CD8 (SK1)	BD Biosciences
V450	IgD (IA6-2)	BD Biosciences	CD127 (BV421)	BD Biosciences
V500	CD3 (UCHT1)	BD Biosciences	CD3 (UCHT1)	BD Biosciences

**Table 4:** The phenotype of lymphocyte subpopulations.

Subpopulation	Phenotype	Subpopulation	Phenotype
B Lymphocytes	CD45++ CD19+ CD3−	Class-Switched BL	CD19+ CD27+ CD38+ IgD− IgM−
BL Naive	CD19+ CD27− IgD+	No Class-Switched BL	CD19+ CD27+ CD38+ IgM+
BL Memory	CD19+ CD27+ CD38+	BL Transitional	CD19+ CD27- CD24+ CD38++ IgM+
BL MZ	CD19+ CD27+ IgD+	Plasmablasts	CD19+ CD27++ CD38++ IgD− IgM−
T Lymphocytes	SSC/FSC low CD3+	T Lymphocytes Memory	CD3+ CD45RO+
T CD4 Lymphocytes	CD3+ CD4+	T Lymphocytes Activated	CD3+ HLA-DR+
T CD8 Lymphocytes	CD3+ CD8+	T regs Lymphocytes	CD3+ CD4+ CD127low CD25++

BL, B Lymphocytes; MZ, Marginal zone; T regs; T regulatory.

## Data Availability

The data presented in this study are openly available at doi.org/10.6084/m9.figshare.14347124.

## Supplementary Materials

The following are available online at https://www.mdpi.com/article/10.3390/diagnostics11040641/s1, Figure S1. Monitoring of subtypes of B lymphocytes in kidney transplant patients. The relative frequencies (black) and the absolute values (blue) of the B lymphocyte subpopulations were represented at pre-transplant, 3 m, and 6 m and compared at three months (3 m) and six months (6 m) post-transplantation to pre-transplantation (pre) using the Wilcoxon test for paired samples. Values of *p* < 0.05 were considered statistically significant. * *p* < 0.05; ** *p* < 0.01; *** *p* < 0.001. Figure S2. Monitoring of B lymphocytes in RTRs suffering acute rejection. The relative frequencies (left axis) and the absolute values (right axis) of the B lymphocyte subpopulations were compared between the NRA (black) and AR (red) groups. The Mann–Whitney U test was used to compare both groups for each follow-up time. Values of *p* < 0.05 were considered statistically significant. * *p* < 0.05. Figure S3. Analysis strategy of B lymphocyte subpopulations. First, lymphocytes were selected from the intersection between the FSC/SSC low and SSC low/CD45++ region. B lymphocytes were defined as those lymphocytes with a CD3− CD19+ phenotype. The different B lymphocyte subpopulations were then defined using CD24, CD27, CD38, IgD, and IgM. Abbreviations: NCS, No Class-Switched; CS, Class-switched; MZ, Marginal zone. Figure S4. T-lymphocyte subpopulation analysis strategy. Lymphocytes were selected in the FSC/SSC low region (Not shown in the figure). T lymphocytes were defined as those lymphocytes with a CD3+ CD19- phenotype. The T lymphocytes were then selected based on the expression of CD4 or CD8. Regulatory T lymphocytes (Treg) were selected as those CD4 T lymphocytes with a CD127lowCD25++ phenotype. Memory and activated T cells were selected based on the expression of CD45RO and HLA-DR, respectively. Table S1. Cluster B lymphocyte levels at pre-transplantation. Table S2. Cluster B lymphocyte levels at three months post-transplant. Table S3. Cluster B lymphocyte levels at six months post-transplant.
