# Supplementary material for: Monitoring of B Cell in Kidney Transplantation: Development of a Novel Clusters Analysis and Role of Transitional B Cells in Transplant Outcome
Source: Diagnostics (Basel). 2021 Apr 1;11(4):641. doi: 10.3390/diagnostics11040641 (PMC8065535; doi:10.3390/diagnostics11040641)
Supplement: Supplementary file 1 [file diagnostics-11-00641-s001.pdf]

Table 1. Cluster B lymphocyte levels at pre-transplantation.

Subpopulations	Cluster			P^a
	A1 (%)	A2 (%)	A3 (%)	
**B Lymphocytes**	6.02±3.01	8.94±5.29	8.60±3.61	0.370
**Naive LB**	52.6±13.9	70.2±5.62	84.6±6.74	<0.001[b,c,d]
**Memory LB**	33.7±12.5	18.3±3.85	7.76±3.15	<0.001[b,c,d]
**LB MZ**	14.5±7.82	7.96±3.83	3.31±1.40	<0.001[c,d]
**LB NCS**	13.7±7.11	7.05±3.22	4.40±3.62	<0.001[b,c]
**LB CS**	17.7±6.32	9.75±4.47	2.95±1.76	<0.001[b,c,d]
**Plasmablasts**	3.02±1.47	1.12±0.74	0.27±0.17	<0.001[b,c,d]
**Transitional LB**	1.42±2.53	0.85±0.74	4.29±7.06	0.176

LB, Lymphocytes B; MZ; Marginal Zone; NCS, No *Class-Switched*; CS, *Class-Switched*. Data expressed as median± standard deviation. [a] Comparisons performed by the Kruskal-Wallis tests. Values $P<0.05$ were considered statistically significant. [b] Differences between cluster A1 and the A2. [c] Differences between cluster A1 and A3. [d] Differences between cluster A2 and the A3.

Table 2. Cluster B lymphocyte levels at three months post-transplant.

Subpopulations	Cluster			P^a
	B1 (%)	B2 (%)	B3 (%)	
**B Lymphocytes**	6.15±4.16	12.4±13.4	7.56±3.68	0.549
**Naive LB**	46.7±8.73	67.9±6.93	68.2±9.05	**<0.001[b,c]**
**Memory LB**	38.5±7.11	21.1±2.50	17.0±6.62	**<0.001[b,c]**
**LB MZ**	16.5±4.82	11.5±4.28	6.43±2.96	**<0.001[c,d]**
**LB NCS**	17.4±4.53	11.1±3.70	6.58±2.85	**<0.001[c]**
**LB CS**	19.6±7.07	8.54±3.45	9.60±4.86	**0.001[b,c]**
**Plasmablasts**	2.31±1.33	2.94±2.14	0.91±0.71	**0.005[c,d]**
**Transitional LB**	0.51±0.69	1.27±0.84	0.37±0.33	**0.012[b,d]**

Abbreviations: LB. Lymphocytes B; MZ; Marginal Zone; NCS. No *Class-Switched*; CS.*Class-Switched*. Data expressed as median± SEM. [a] Comparisons performed by the Kruskal-Wallis tests. [b] Differences between cluster B1 and the B2. [d] Differences between cluster B1 and the B3. [d] Differences between cluster B2 and the B3. Values $P<0.05$ were considered statistically significant and indicated in bold.

Table 3. Cluster B lymphocyte levels at six months post-transplant.

Subpopulations	Cluster			P^a
	C1 (%)	C2 (%)	C3 (%)	
**B Lymphocytes**	4.63±2.17	4.05±2.09	8.47±4.67	**0.008 [c,d]**
**Naive LB**	48.9±13.0	57.4±9.36	72.0±8.13	**<0.001[c,d]**
**Memory LB**	36.6±10.1	23.3±4.87	16.5±3.55	**<0.001[c]**
**LB MZ**	19.0±4.62	6.40±2.65	8.04±2.60	**<0.001[b,c]**
**LB NCS**	18.0±4.00	5.89±2.38	7.99±3.00	**<0.001[b,c]**
**LB CS**	15.9±9.90	16.1±5.06	7.72±3.55	**0.001[c,d]**
**Plasmablasts**	1.93±2.70	2.76±1.46	0.95±0.74	**0.005 [d]**
**Transitional LB**	0.56±0.72	1.56±2.47	0.30±0.35	0.593

LB. Lymphocytes B; MZ; Marginal Zone; NCS. No *Class-Switched*; CS.*Class-Switched*. Data expressed as median± SEM. [a] Comparisons performed by the Kruskal-Wallis test. Values $p<0.05$ are considered statistically significant and are indicated in bold. [b] Differences between the cluster C1 and the C2. [c]Differences between cluster C1 and the C3. [d] Differences between the cluster C2 and the C3.

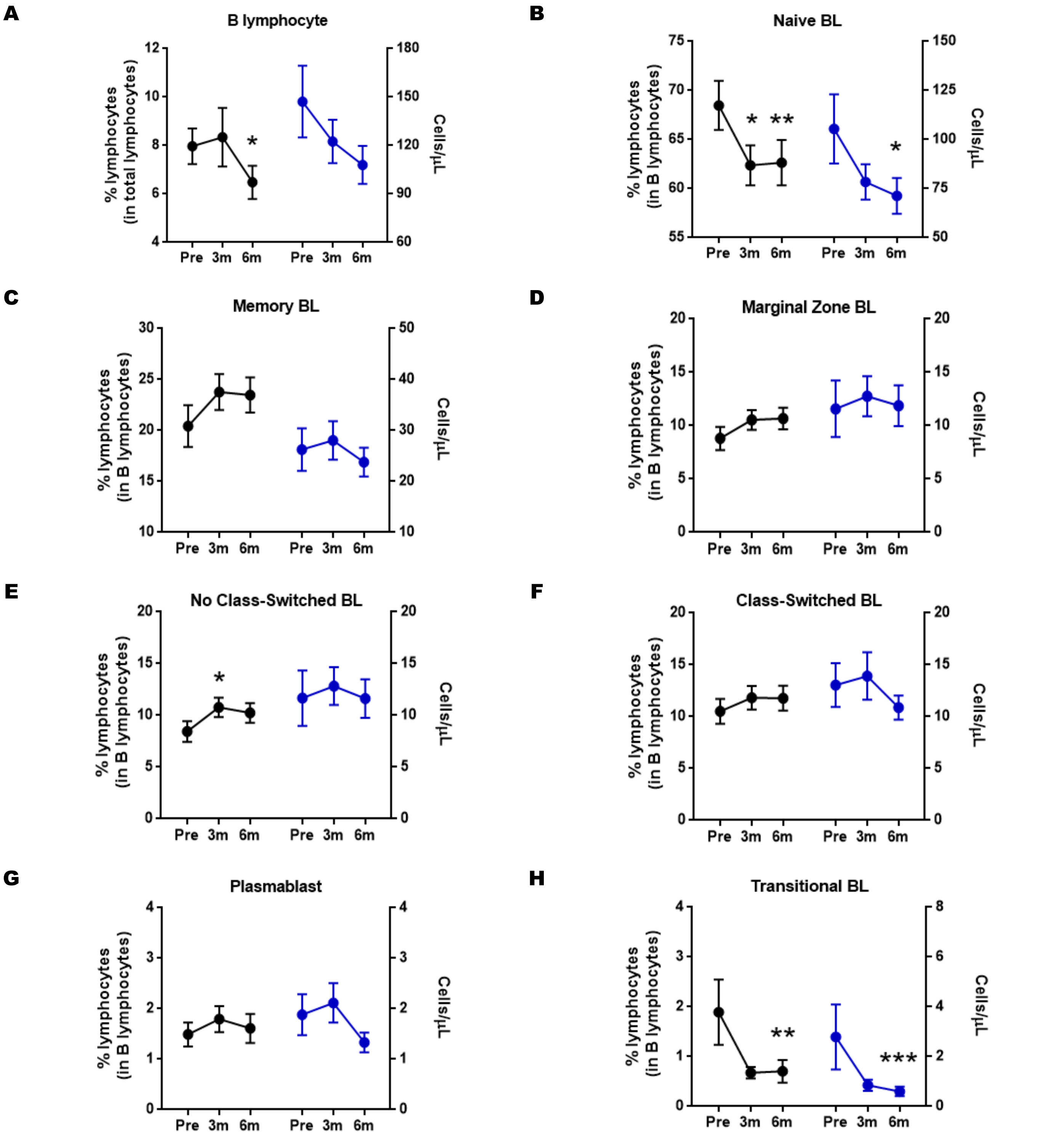

Figure 1. Monitoring of subtypes of B lymphocytes in kidney transplant patients. The relative frequencies (black) and the absolute values (blue) of the B lymphocyte subpopulations were rep-resented at pre-transplant, 3 m, and 6 m and compared at three months (3 m) and six months (6 m) post-transplantation to pre-transplantation (pre) using the Wilcoxon test for paired samples. Values of $p < 0.05$ were considered statistically significant. * $p < 0.05$; ** $p < 0.01$; *** $p < 0.001$.

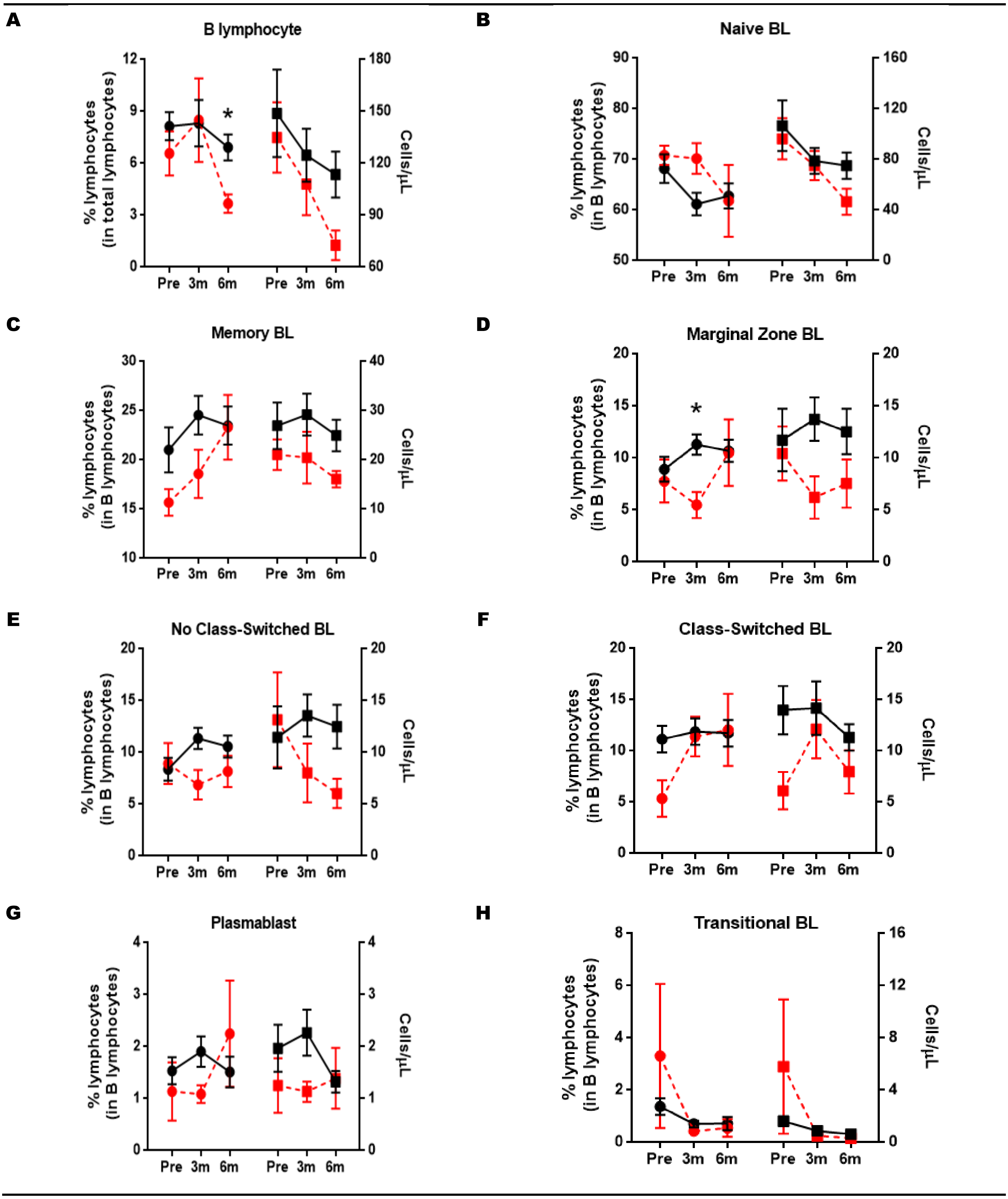

Figure S2. Monitoring of B lymphocytes in RTRs suffering acute rejection. The relative frequencies (left axis) and Table 0. were considered statistically significant. * $p < 0.05$.

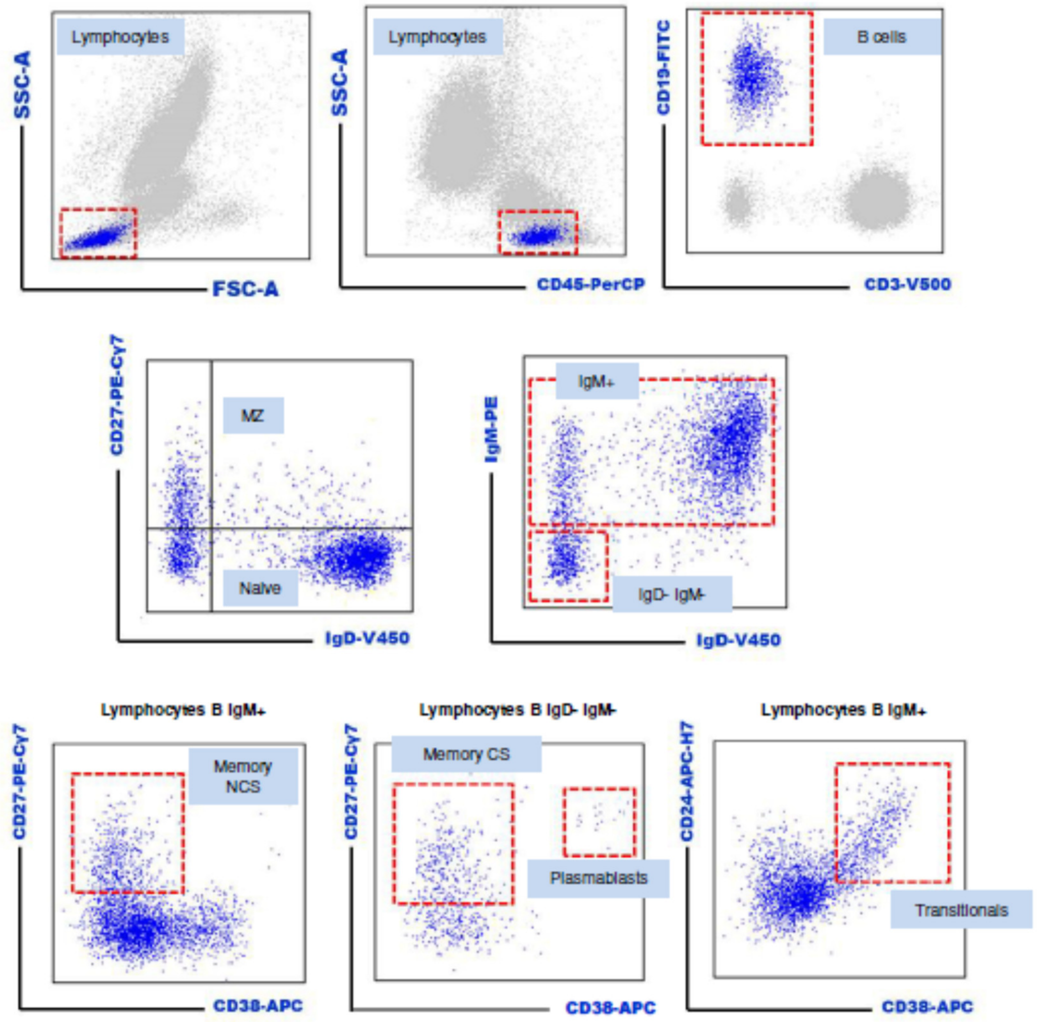

Figure 3. Analysis strategy of B lymphocyte subpopulations. First, lymphocytes were selected from the in-tersection between the FSC/SSC low and SSC low/CD45++ region. B lymphocytes were defined as those lymphocytes with a CD3− CD19+ phenotype. The different B lymphocyte subpopulations were then defined using CD24, CD27, CD38, IgD, and IgM. Abbreviations: NCS, No Class-Switched; CS, Class-switched; MZ, Marginal zone.

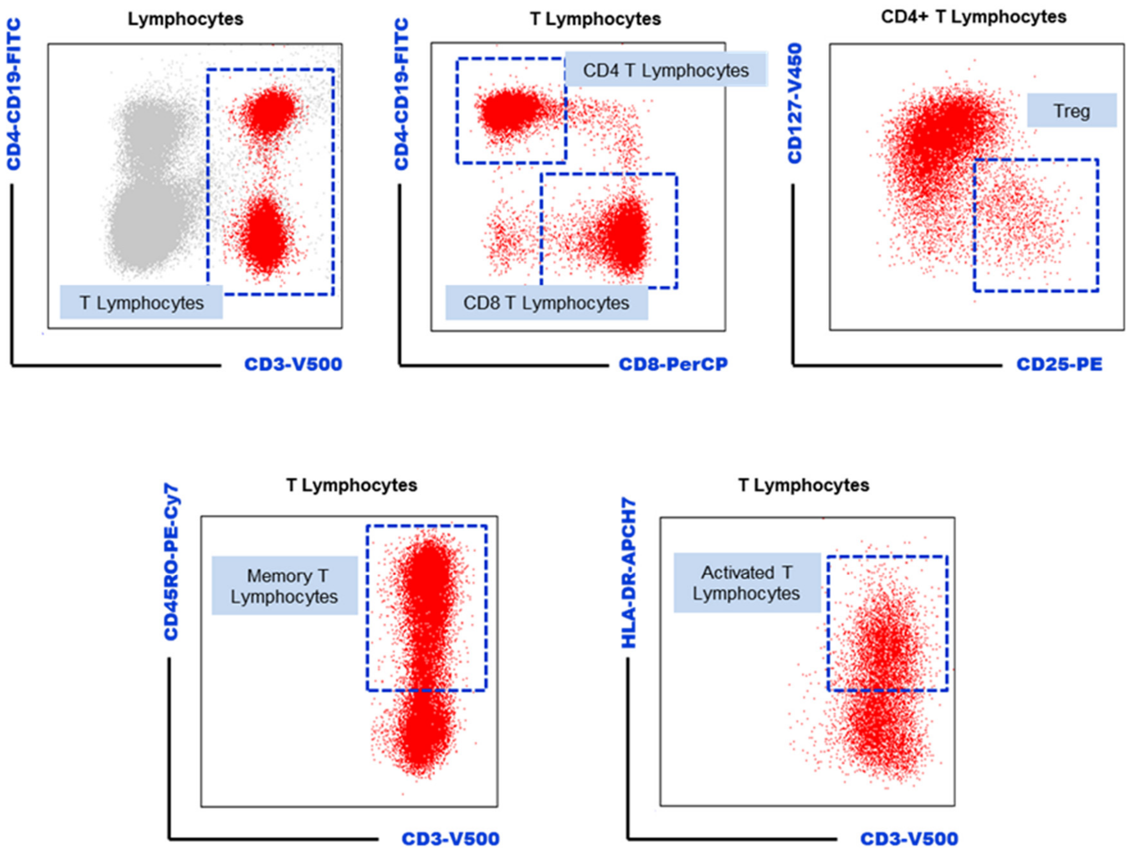

Figure 4. T-lymphocyte subpopulation analysis strategy. Lymphocytes were selected in the FSC/SSC low region (Not shown in the figure). T lymphocytes were defined as those lymphocytes with a CD3+ CD19- phenotype. The T lym-phocytes were then selected based on the expression of CD4 or CD8. Regulatory T lymphocytes (Treg) were selected as those CD4 T lymphocytes with a CD127lowCD25++ phenotype. Memory and activated T cells were selected based on the expression of CD45RO and HLA-DR, respectively.
